# Exactly solvable spin chain models corresponding to BDI class of topological superconductors

**DOI:** 10.1038/srep32720

**Published:** 2016-09-06

**Authors:** S. A. Jafari, Farhad Shahbazi

**Affiliations:** 1 Department of Physics, Sharif University of Technology, Tehran 11155-9161, Iran; 2 Department of Physics, Isfahan University of Technology, Isfahan 84156-83111, Iran; 3 School of Physics, Institute for Research in Fundamental Sciences, Tehran 19395-5531, Iran; 4 Center of excellence for Complex Systems and Condensed Matter (CSCM), Sharif University of Technology, Tehran 1458889694, Iran

## Abstract

We present an exactly solvable extension of the quantum XY chain with longer range multi-spin interactions. Topological phase transitions of the model are classified in terms of the number of Majorana zero modes, *n*_*M*_ which are in turn related to an integer winding number, *n*_*W*_. The present class of exactly solvable models belong to the BDI class in the Altland-Zirnbauer classification of topological superconductors. We show that time reversal symmetry of the spin variables translates into a *sliding* particle-hole (PH) transformation in the language of Jordan-Wigner fermions – a PH transformation followed by a *π* shift in the wave vector which we call it the *π*PH. Presence of *π*PH symmetry restricts the *n*_*W*_ (*n*_*M*_) of time-reversal symmetric extensions of XY to odd (even) integers. The *π*PH operator may serve in further detailed classification of topological superconductors in higher dimensions as well.

Majorana fermion (MF) is a particle whose antiparticle is itself^1^. So far, All attempts to find such an elementary particles in nature have been unsuccessful. However, condensed matter systems provide promising ground for the emergence of MF’s as quasiparticle excitations. Normally, any fermion can be split into a real and imaginary parts, each being a MF in literally the same way that a complex variable can be written in terms of real and imaginary part. However the deal is that some Hamiltonians in condensed matter allow to localize MFs in different regions of the space. In this case the whole fermionic state is topologically protected against the effect of local perturbations on each localized MF. The stability of MF’s versus local disturbances makes this states ideal for low-decoherence quantum computing.

The Bogoliubov excitations in superconductors are the superpositions of electrons and holes, hence these quasiparticles could be candidates for the realization MF’s. The MF’s property of being its own antiparticle rules out the s-wave superconductors as the host of such quasiparticles, since the Bogolons in these type of superconductors are the superposition of electrons and holes with opposite spins. However, a one-dimensional (1D) spin-less superconductor with p-wave symmetry could be such a candidate, as initially proposed by Kitaev^2^. MF’s appear in 1D Kitaev’s chain as the localized zero energy modes at the chain-ends.

Interestingly, it can be seen that a 1D quantum Ising model in a transverse field can be mapped to a Kitaev’s chain using Jordan-Wigner (JW) transformations^3^, indicating the topological features of the Ising spin chain. In this work, we introduce a class of exactly solvable 1D spin chains with specific type of interactions, which incorporate the topological properties leading to presence of an arbitrary number of MF end-modes depending on the range of the interactions.

The spectrum of Quantum XY model is exhausted by the emergent Jordan-Wigner (JW) fermions^3^,^4^. The anisotropy of the exchange coupling generates p-wave superconducting pairing between spinless JW fermions leading to unpaired MF at ends of an open chain^2^. Adding further neighbor XY couplings in general spoils the exact solvability because the JW transformation incorporates appropriate (non-local) phase strings in order to fulfill anti-commutation algebra^3^. In the context of the Ising in a transverse field (ITF) model it was recently shown that adding appropriately engineered three-spin interactions can still leave it exactly solvable^5^. Given that ITF and XY model are related by a duality transformation^6^,^7^ we expect similar extensions to work for the XY model. In this letter we classify generalizations of the XY model with arbitrary *n*-spin interactions in terms of a *π*PH symmetry that is a PH transformation followed by a sign alternation in one sublattice. We show that in presence of *π*PH corresponding to every MF there will be a partner MF which will correspondingly restrict the possible winding numbers. Let us start with the XY Hamiltonian,


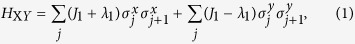


to which we add a *n*-spin interaction,


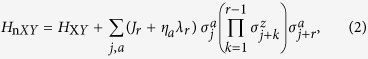


where *a* = *x*, *y* and *η*_*x*_ = −*η*_*y*_ = 1. Here *r* = *n* − 1 denotes the range of *n*-spin interaction. *J*_*r*_ is the longer range exchange and *λ*_*r*_ denotes the longer range XY anisotropy. For this Hamiltonian (nXY model) the quantity 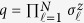 is a constant of motion. Two possible *q* = ±1 values correspond to number parity of JW fermions and hence the above generalization is expected to give a superconducting system. Indeed the JW transformation^3^,


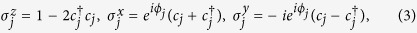


where *ϕ*_*j*_ is the phase string defined as 
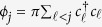
 converts the above Hamiltonian to,


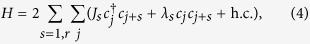


where longer range exchange and anisotropy parameter *J*_*s*_,*λ*_*s*_ give rise to hopping and pairing between *s*’th neighbors, respectively. Note the important role played by *σ*^*z*^ phases is to cancel the unwanted JW phases which renders the nXY Hamiltonian to the quadratic form (4).

For even (odd) *r* the generalized term involves *n* = *r* + 1 spins which will be odd (even) under the time-reversal (TR). Let us now figure out how does the JW dictionary translate the TR operation of spin variables. The TR changes the sign of spin operators 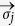. Sign reversal of 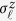 with 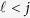 implies that under TR the non-local phase string is transformed as 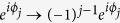. Therefore the TR of spins for JW fermions translates to,


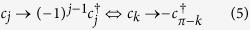


which is nothing but the PH transformation followed a *π* shift in the *k*-space – or *sliding* PH – that will be denoted by *π*PH in this paper. The *π*PH can further resolve the topological classification of the Altland-Zirnbauer (AZ) classification of topological superconductors^8^ that are based on PH, TR, and sublattice symmetries^9^,^10^,^11^,^12^. In the following we discuss in detail two prototypical cases corresponding to longer range interaction with range *r* = 2, 3 involving *n* = 3, 4 spins, respectively. Then we present general arguments for arbitrary *r* and discuss an even-odd dichotomy related to the *π*PH transformation.

## Results

### 3XY Model

In this case the *k*-space representation of the JW Hamiltonian is,


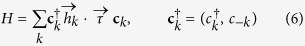


where *τ*^*a*^, *a* = *x*, *y*, *z* stands for Pauli matrices in the Nambu space and 
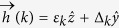
 with


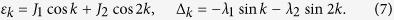


Note that pairing with (hopping to) a neighbor at distance *r* = 2 has added a *λ*_2_sin 2*k* (*J*_2_cos 2*k*) term to the Anderson pseudovector 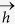 representation of the Hamiltonian matrix. This general feature holds for any *r*. The eigenvalues and eigenvectors of 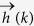 are given by,


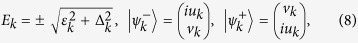


where the coherence factors are parameterized in terms of a phase 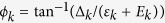 as *u*_*k*_ = sin(*ϕ*_*k*_/2) and *v*_*k*_ = cos(*ϕ*_*k*_/2). The JW Hamiltonian (6) is diagonalized in terms of Bogolons 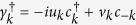.

**Figure 1:**
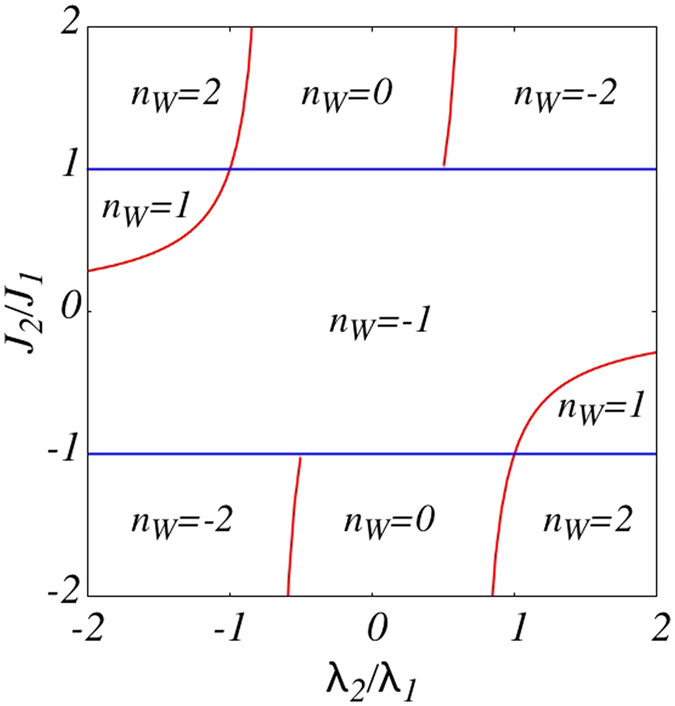
The phase diagram of 3XY model in parameter space. The phase boundary curves correspond to gap closing separating topologically distinct phases characterized by a winding number *n*_*W*_ as indicated in the figure.

**Figure 2:**
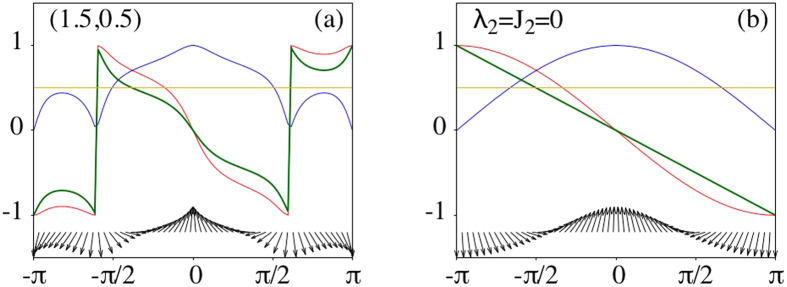
Wave function and winding pattern for representative points in regions various phases of Fig. 1. The red and blue curves represent the coherence factors *u*_*k*_ and *v*_*k*_ as a function of *k* and the green curve corresponds to the *ϕ*_*k*_/*π*. Black arrows are unit vectors constructed from Anderson pseudovector (Δ_*k*_, *ε*_*k*_) at every *k* in the 1BZ. The coordinate (*λ*_2_/*λ*_1_, *J*_2_/*J*_1_) is shown in the top left of each panel. The resulting *n*_*W*_ is used to label regions of Fig. 1.

The boundaries of the phase diagram of the 3XY model can be analytically calculated by investigating the gap closing of the spectrum (8) that happens when both *ε*_*k*_ and Δ_*k*_ vanish which gives following equations for the phase boundaries,


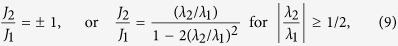


which has been plotted in Fig. 1. It is remarkable that the phase boundary is given in terms of ratios *J*_2_/*J*_1_ and *λ*_2_/*λ*_1_. This property is also general and the phase diagram is determined only in terms of the ratios *J*_*r*_/*J*_1_ and *λ*_*r*_/*λ*_1_. In Fig. 1 each region is labeled by a winding number *n*_*W*_. This topological invariant corresponds to the number of times the unit circle is covered by the vector (Δ_*k*_, *ε*_*k*_) as *k* varies across the first Brillouin zone (1BZ)^13^. These vectors are represented by black arrows in Fig. 2 which correspond to green curve representing *ϕ*_*k*_/*π*. The winding pattern of arrows and the global variation profile of *ϕ*_*k*_ does not change as long as the phase boundaries of Fig. 1 are not crossed. This means that *n*_*W*_ is a topological invariant^14^. For the 3XY model five possible values *n*_*W*_ = 0, ±1, ±2 can be extracted from analysis similar to Fig. 2. The resulting *n*_*W*_ values are used to label regions of Fig. 1. The phase with *n*_*W*_ = 0 is adiabatically connected to the trivially gapped phase that can be reached by an applied field *h* → ±∞ that couples to *σ*^*z*^ without gap closing. This makes the *n*_*W*_ = 0 region topologically trivial. This is while the other phases with *n*_*W*_ ≠ 0 are separated from the trivially gapped phase by a gap closing. The panel (b) of Fig. 2 corresponds to *λ*_2_ = *J*_2_ = 0 where *λ*_1_ ≠ 0 is adiabatically connected to the Ising limit *λ*_1_ = 1. This is why the phase boundary (9) is determined by the ratio *λ*_2_/*λ*_1_.

Let us show that for any *r* the nXY model falls into BDI class^10^ which in turn allows for winding number classification. First let us check the TR symmetry. For spinless fermions TR operator *T* is simply a complex conjugation. Using the Nambu space representation, Eq. (6), we have 
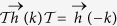
 which represents the TR symmetry of the JW Hamiltonian. Now defining the operator 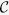 = *τ*_*x*_*K* as the PH transformation, one finds 
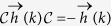
 that checks *the* PH symmetry. Finally for the chiral symmetry we have, 
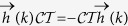
. Let us emphasize that for every *r*, only sin(*rk*) functions appear in the pairing term and hence the above properties that rest on odd parity of Δ_*k*_ apply to nXY model. Since for the present spinless JW fermions one has 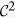 = 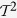
= +1, the nXY belongs to BDI class in the AZ classification of topological superconductors and hence allows for integer (winding number) classification of the topological phases. However both range of possible integers and whether they are even or odd is determined by *r* which in turn is connected to the presence or absence of the sliding PH symmetry, *π*PH. To set the stages for discussion of arbitrary *r*, let us consider the next prototypical case of 4-spin interactions.

### 4XY Model

**Figure 3:**
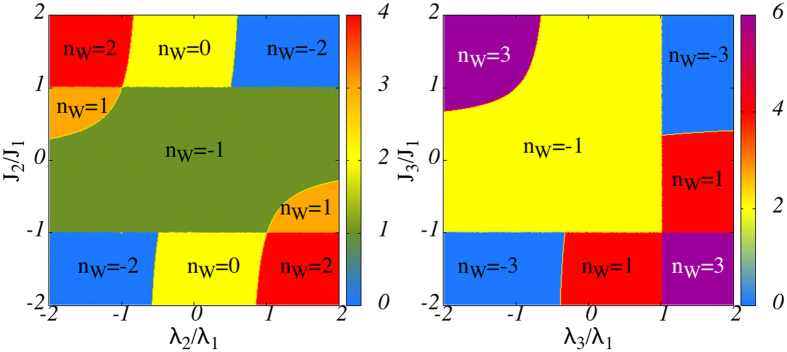
Color map of the number of MFs, *n*_*M*_ in the parameter space for two extensions 3XY (left) and 4XY (right) corresponding to spin clusters of range *r* = 2, 3, respectively. The number *n*_*M*_ of MFs can be read from the legend to the right of each figure and the winding number *n*_*W*_ has been calculated and used to label each region in the figure. The 4XY model has *π*PH symmetry in JW representation which restricts *n*_*M*_ to even values and *n*_*W*_ to odd values only. Obviously the origin in both cases correspond to *r* = 1 which is not continously connected to topologically distinct cases of *r* = 2 (left) nor *r* = 3 (right).

The four-spin term preserves the TR symmetry of the spin model. The components of Anderson pseudovector are given by, *ε*_*k*_ = *J*_1_cos(*k*) + *J*_3_cos(3*k*) and Δ_*k*_ = *λ*_1_sin(*k*) + *λ*_3_sin(3*k*). The phase boundaries will be given by setting *ε*_*k*_ = Δ_*k*_ = 0. Therefore the gap closing curves of this model are the lines *λ*_3_/*λ*_1_ = 1 and *J*_3_/*J*_1_ = −1, and the curve


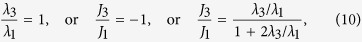


for *λ*_3_/*λ*_1_ ≥ 1 and *λ*_3_/*λ*_1_ ≤ −1/3. These curves partition the the parameter space (*λ*_3_/*λ*_1_, *J*_3_/*J*_1_) into seven regions represented in the right panel of Fig. 3 each characterized by *n*_*W*_ = ±1, ±3. The left panel of this figure represents same set of data for 3XY model. In both *r* = 2, 3 cases |*n*_*W*_| ≤ *r* while for odd *r* only odd values of *n*_*W*_ are picked. To explain the meaning of the color code in this figure, let us discuss the number *n*_*M*_ of MFs for a general *r*.

### Majorana end modes

To further understand the properties of nXY model, let us now consider an open nXY chain and discuss the Majorana zero modes of the chain. Presence of MFs requires equal spin (or spinless pairing)^13^,^15^ which engineered in one^16^ or two^17^ dimensions. In the case of XY models the spinless pairing *emerges*^2^. Let us now see how do MFs appear in nXY model. In terms of MFs 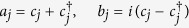 the nXY model becomes,


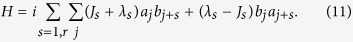


Varrying the above Hamiltonian the following wave equation is obtained:


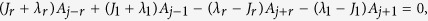


where the zero in the right hand side appears because we are searching for zero energy solutions. If we search for MFs of type *a* localized near the origin for the nXY chain, the amplitudes *B*_*j*_ for every site *j* are zero and hence the wave function (*A*_1_, *B*_1_, …, *A*_*N*_, *B*_*N*_) reduces to, (*A*_1_, 0, *A*_2_, …, *A*_*N*_, 0).

For the ansatz of the form *A*_*j*_ ~ *x*^*j*^ for the amplitude of MF wave function at site *j* we obtain,


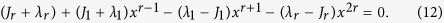


If we searcher for solutions of type (0, *B*_1_, …, 0, *B*_*N*_), we would obtain a similar equation but with *x* → *x*^−1^. To have a normalizable Majorana zero mode of type *a* we need solutions that satisfy |*x*| < 1. Figure 3 represents a color map of the number *n*_*M*_ of MFs of type *a* localized in one end for *r* = 2, 3. The first thing to note is that the phase boundaries in the 3XY model obtained from the MF counting analysis precisely coincides with that in Fig. 1. This is also true for the 4XY model, and the boundaries given by Eq. (10) precisely coincide with that in the right panel of Fig. 3. The color code in each panel of this figure indicates the number of MFs of type *a* bound to left end. For each panel we have explicitly indicated *n*_*W*_. It can observed that in both cases,


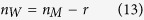


where *r* is the range of interaction. Let us present a heuristic argument that the above formula holds for any *r*.

To proceed further let us first elucidate the meaning of *π*PH in the language of MFs: If we represent the JW “electron” and “hole” operators as 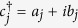 and 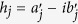 and if we search for MFs with vanishing *b*, *b*′ component, then every zero mode solution (*A*_1_, 0, *A*_2_, 0, …) is mapped by *π*PH to a *partner* MF 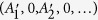 with 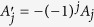.

For even values of *r* where odd number of spin variables are added to the XY model, consider any point in the phase diagram (see left panel of Fig. 3) with given number *n*_*M*_ of MFs. Obviously the generalized *r* + 1-spin interaction breaks TR symmetry. In this case the overall minus arising from TR transformation can be absorbed by the transformation (*λ*_*r*_, *J*_*r*_) → −(*λ*_*r*_, *J*_*r*_). This means that for every MF at point (*λ*_1_, *J*_1_, *λ*_*r*_, *J*_*r*_) of the phase diagram, its partner MF corresponds to point (*λ*_1_, *J*_1_, −*λ*_*r*_, −*J*_*r*_). This explains the inversion symmetry in the left panel of Fig. 3. This can also be seen from Eq. (12) that maps to itself under simultaneous change of *x* → −*x* and (*λ*_*r*_, *J*_*r*_) → −(*λ*_*r*_, *J*_*r*_). For odd values of *r*, there are even number of spins giving a TR symmetric term and hence the TR operation does not produce any minus sign in the *n*-spin term. Therefore corresponding to every MF at any point (*λ*_1_, *J*_1_,*λ*_*r*_, *J*_*r*_), the partner MF also exists at the same point in the parameter space. This can also be seen directly from Eq. (12): Although in general Eq. (12) admits 2*r* solutions such that the number *n*_*M*_ of them satisfying |*x*| < 1 is 0 ≤ *n*_*M*_ ≤ 2*r*. However, for odd *r* this equation becomes an equation of degree *r* in terms of *X* = *x*^2^ which implies that the solutions always come in pairs ±*x* giving partner MFs as *A*_*j*_ ~ (±*x*)^*j*^. This explains why in the right panel of Fig. 3 only colors corresponding to even *n*_*M*_ appear. The presence of *π*PH for odd *r* implies that corresponding to every MF at the chain end, its partner obtained by sign alternation in one-sublattice is also acceptable solution and hence *n*_*M*_ is always even. Now let us discuss how does *π*PH restrict *n*_*W*_.

Consider an arbitrary point in the phase diagram corresponding to pairing amplitudes {*λ*_*s*_}. Since the resulting JW Hamiltonian (4) is TR symmetric, the action of TR is simply *i* → −*i* which can be absorbed by {*λ*_*s*_} → {−*λ*_*s*_}. The later on other hand amounts to changing the sing of the horizontal component Δ_*k*_ of the Anderson pseudovector. Therefore it is the implication of TR symmetry of JW Hamiltonian that corresponding to every winding number *n*_*W*_, there is a winding number −*n*_*W*_ irrespective of whether *r* is even or odd. Now let us argue that the possible values of *n*_*W*_ are bounded by the range *r* of interaction. Every time Δ_*k*_ vanishes, the pseudovector points vertically either to the south or north pole. For the nXY model the maximum number of the zeros of Δ_*k*_ in the 1BZ produced by combination first and *r*’th harmonics of 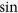 function is *r* which implies |*n*_*W*_| ≤ *r*.

To see when the maximum number of zeros in Δ_*k*_ are realized, it is enough to consider the limit *J*_*r*_ ~ *rλ*_*r*_ ≫ *λ*_1_ ~ *J*_1_ where there are 2*r* + 1 zeros for Δ_*k*_ in the 1BZ which give maximal winding |*n*_*W*_| = *r*. In this limit the secular equation (12) reduces to *x*^−2*r*^ = (*r* − 1)/(*r* + 1) the absolute value of which is always less than unity for every *r* > 0 which realizes maximum number of MFs equal to 2*r*. This means that the maximum values of *n*_*W*_ and *n*_*M*_ happen in the same limit. Now the point with minimum *n*_*W*_ is the TR of the maximal *n*_*W*_. The TR for JW Hamiltonian is equivalent to {*λ*_*s*_} → {−*λ*_*s*_} which is a *π*/2 rotation around the *z*-axis for spin variables and the operation *a* ↔ *b* for MFs which essentially exchanges *x* ↔ *x*^−1^ and hence mapping every state with *n*_*M*_ MFs to a state with 2*r* − *n*_*M*_ MFs. Therefore both upper and lower bounds of 2*r* + 1 possible integers |*n*_*W*_| ≤ *r* and 0 ≤ *n*_*M*_ ≤ 2*r* describe the same physical state. Finally, since both *n*_*M*_ and *n*_*W*_ are unique topological labels of the same state, the mapping between the two sets must be one-to-one. Since the ends of two chains of integers map to each other, we heuristically expect Eq. (13) to hold for any *r*.

Now let us discuss why for odd values of *r* = 2*p* + 1 the *n*_*W*_ is always odd. The *n*_*W*_ changes by half between each two consecutive zeros of Δ_*k*_ = *λ*_1_sin*k* + *λ*_*r*_sin((2*p* + 1)*k*). Suppose that this gap function vanishes at some point k_*_ in the 1BZ. By *π*PH symmetry, relation (5) it also vanishes at *π* − k_*_. The sign of the vertical component *ε*_*k*_ at k_*_ and *π* − k_*_ are opposite as the 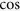 functions appearing in vertical component *ε*_*k*_ of Anderson pseudovector change sign upon going from k_*_ to *π* − k_*_ when *r* is odd.

Starting from the XY model (*p* = 0) and focusing only in the right half of 1BZ with *k* > 0, at *k* = 0 and *k* = *π* the Anderson vectors point to north and south poles respectively (Fig. 2b) which means that in the right half of 1BZ one picks up a half-integer winding. For every k_*_ if the winding vector points to some pole the one at *π* − k_*_ will point to opposite pole, corresponding to every pair of roots k_*_, *π* − k_*_ of Δ_*k*_, an integer winding is inserted to the right half of 1BZ. This means that always half-integer windings are possible in the right half of 1BZ. Therefore the winding number picked over the whole 1BZ is an odd number. That is why in the right panel of Fig. 3 we only have odd winding numbers. The fact that for odd *r*, only even number of MFs and only odd *n*_*W*_s are possible is consistent with Eq. (13). This line of reasoning implies that in the simple case *p* = 0 corresponding to XY (or Kitaev) chain, by *π*PH symmetry the topologically non-trivial phase always hosts two independent Majorana end modes related by *A*_*j*_ ↔ −(−1)^*j*^*A*_*j*_.

It can be noticed that the *Z*_2_ index defined by *ν* = sign(*ε*_0_)sign(*ε*_*π*_)^13^, gives *ν* = −1 when *r* is odd. However, when *r* is even, *ν* = sign(|*J*_*r*_/*J*_1_| − 1). For even *r*, the *ν* = +1(−1) corresponds to even (odd) values of *n*_*W*_. The physical interpretation of *ν* is as follows: When *ν* = +1(−1) the identity of Bogolons does not change (changes) from hole-like to particle-like when *k* spans the range [0, *π*]. The presence of *π*PH symmetry for odd *r* guarantees that the above *Z*_2_ index takes only one value −1 which means that the charge character of Bogolons at *k* = 0 and *k* = *π* are opposite. Breaking *π*PH allows for both ±1 values.

## Discussion

We have presented an exactly solvable extension of the quantum XY model that involves clusters of *n* = *r* + 1 spins interacting at range *r*. The ensuing JW representation is a topological superconductor in BDI class. We showed that the TR operation of original spin variables translates to a sliding PH transformation of JW fermions, *π*PH. The presence of *π*PH implies that corresponding to every MF wave function *A*_*j*_, there is a partner MF whose wave function is −(−1)^*j*^*A*_*j*_ which in turn restricts the number of MFs to even values only. The *π*PH also implies that the roots of pairing potential come in pairs which restricts the *Z* winding numbers to odd integers only. Let us discuss the stability of MFs. If we perturb the nXY Hamiltonian with a generic 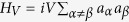 where *α*, *β* run over the number MFs with *V* a real number in order to gurantee Hermiticity of *H*_*V*_. Such a perturbation can in principle lift the degeneracy and push the localized modes away from zero energy. However, *H*_*V*_ is odd under TR and charge conjugation. Therefore the symmetry preserving pertubations are not able to destroy the zero modes.

The Bott periodicity^9^ implies that there should exist similar restriction in higher dimensions on topological invariants when *π*PH is a symmetry. It will be interesting to study possible higher dimensional models with *π*PH symmetry in electronic systems^17^,^18^,^19^. The number *n*_*M*_ of MFs leaves a unique signature in tunneling experiments^20^ and hence remains directly accessible to experiments. Array of magnetic nano-particles on a superconductor is described by an effective theory that includes *r* = 2 hopping between the spinless fermions^12^,^21^ which may serve as potential platform to materialize 3XY model.

## Methods

In the fermionization of the starting spin model we have used the Jordan-Wigner transformation. In finding the wave functions we have used the ansatz based on Z-transform method which eventually leads to polynomial equation that has been solved with Mathematica software.

## Additional Information

**How to cite this article**: Jafari, S. A. and Shahbazi, F. Exactly solvable spin chain models corresponding to BDI class of topological superconductors. *Sci. Rep.* **6**, 32720; doi: 10.1038/srep32720 (2016).
