# Corrigendum: Exactly solvable spin chain models corresponding to BDI class of topological superconductors

**DOI:** 10.1038/srep35124

**Published:** 2016-10-19

**Authors:** S. A. Jafari, Farhad Shahbazi

Scientific Reports 6: Article number: 3272010.1038/srep32720; published online: 09 06 2016; updated: 10 19 2016

In the original version of this Article, S.A. Jafari was incorrectly affiliated with ‘Department of Physics, Isfahan University of Technology, Isfahan 84156-83111, Iran’. The correct affiliations are listed below:

Department of Physics, Sharif University of Technology, Tehran 11155-9161, Iran.

Center of excellence for Complex Systems and Condensed Matter (CSCM), Sharif University of Technology, Tehran 1458889694, Iran.

School of Physics, Institute for Research in Fundamental Sciences, Tehran 19395-5531, Iran.

In addition, Farhad Shahbazi was incorrectly affiliated with ‘Center of excellence for Complex Systems and Condensed Matter (CSCM), Sharif University of Technology, Tehran 1458889694, Iran’. The correct affiliations are listed below:

Department of Physics, Isfahan University of Technology, Isfahan 84156-83111, Iran.

School of Physics, Institute for Research in Fundamental Sciences, Tehran 19395-5531, Iran.

These errors have been corrected in both the HTML and PDF versions of the Article.

